# Investigation of artificial intelligence integrated fluorescence endoscopy image analysis with indocyanine green for interpretation of precancerous lesions in colon cancer

**DOI:** 10.1371/journal.pone.0286189

**Published:** 2023-05-25

**Authors:** Jinhyeon Kim, Hajung Kim, Yong Sik Yoon, Chan Wook Kim, Seung-Mo Hong, Sungjee Kim, Doowon Choi, Jihyun Chun, Seung Wook Hong, Sung Wook Hwang, Sang Hyoung Park, Dong-Hoon Yang, Byong Duk Ye, Jeong-Sik Byeon, Suk-Kyun Yang, Sun Young Kim, Seung-Jae Myung

**Affiliations:** 1 Digestive Diseases Research Center, University of Ulsan College of Medicine, Seoul, Republic of Korea; 2 Convergence Medicine Research Center, Asan Medical Center, Seoul, Republic of Korea; 3 Department of Gastroenterology, Asan Medical Center, University of Ulsan College of Medicine, Seoul, Republic of Korea; 4 Department of Colon and Rectal Surgery, Asan Medical Center, University of Ulsan College of Medicine, Seoul, Republic of Korea; 5 Department of Pathology, Asan Medical Center, University of Ulsan College of Medicine, Seoul, Republic of Korea; 6 Department of Chemistry and School of Interdisciplinary Bioscience and Bioengineering, Pohang University of Science & Technology, Pohang, Gyeongbuk, Republic of Korea; 7 School of Interdisciplinary Bioscience and Bioengineering, Pohang University of Science & Technology, Pohang, Gyeongbuk, Republic of Korea; 8 Asan Institute for Life Sciences, Asan Medical Center, University of Ulsan College of Medicine, Seoul, Republic of Korea; 9 Edis Biotech, Songpa-gu, Seoul, Republic of Korea; Osaka International Cancer Institute: Osaka Kokusai Gan Center, JAPAN

## Abstract

Indocyanine green (ICG) has been used in clinical practice for more than 40 years and its safety and preferential accumulation in tumors has been reported for various tumor types, including colon cancer. However, reports on clinical assessments of ICG-based molecular endoscopy imaging for precancerous lesions are scarce. We determined visualization ability of ICG fluorescence endoscopy in colitis-associated colon cancer using 30 lesions from an azoxymethane/dextran sulfate sodium (AOM/DSS) mouse model and 16 colon cancer patient tissue-samples. With a total of 60 images (optical, fluorescence) obtained during endoscopy observation of mouse colon cancer, we used deep learning network to predict four classes (Normal, Dysplasia, Adenoma, and Carcinoma) of colorectal cancer development. ICG could detect 100% of carcinoma, 90% of adenoma, and 57% of dysplasia, with little background signal at 30 min after injection via real-time fluorescence endoscopy. Correlation analysis with immunohistochemistry revealed a positive correlation of ICG with inducible nitric oxide synthase (iNOS; r > 0.5). Increased expression of iNOS resulted in increased levels of cellular nitric oxide in cancer cells compared to that in normal cells, which was related to the inhibition of drug efflux via the ABCB1 transporter down-regulation resulting in delayed retention of intracellular ICG. With artificial intelligence training, the accuracy of image classification into four classes using data sets, such as fluorescence, optical, and fluorescence/optical images was assessed. Fluorescence images obtained the highest accuracy (AUC of 0.8125) than optical and fluorescence/optical images (AUC of 0.75 and 0.6667, respectively). These findings highlight the clinical feasibility of ICG as a detector of precancerous lesions in real-time fluorescence endoscopy with artificial intelligence training and suggest that the mechanism of ICG retention in cancer cells is related to intracellular nitric oxide concentration.

## Introduction

Colorectal cancer (CRC) is the most common cancer worldwide and the leading cause of cancer-related deaths [1]. The progression of adenoma leads to CRC, and involves the conventional tubular adenoma and alternative serrated polyp pathways [2]. Chronic inflammation is a major risk factor that induces different morphologies and dysplasia-carcinoma progression in patients with inflammatory bowel disease (IBD) [3]. Hence, the detection of early phase cancer or dysplasia as precancerous lesions could lead to decreased mortality.

Currently, white-light endoscopy is the gold standard for screening colonoscopy; however, the miss rate of adenomas during endoscopy is 24.1% and for smaller and flat or sessile polyps it is significantly higher [4]. For sensitive diagnosis of CRC lesions, advanced molecular imaging techniques, such as fluorescence endoscopy, which targets tumor-specific molecules, have been developed [5–7]. Fluorescence-based tumor imaging can be a useful technique for early cancer detection owing to its advantageous high specificity and sensitivity, real-time imaging, and absence of radiation exposure [8]. In previous studies, a fluorescence-labeled compound, which could bind to tumor-specific molecules, such as vascular endothelial growth factor (VEGF) [9, 10], epidermal growth factor receptor (EGFR) [11], colon cancer specific protein-2 (CCSP-2) [12], endothelial A receptor (ET_A_R) [13], and c-Met [14] was developed to detect colon cancer. Although various probes have been used, their safety in the human body and their ability to detect precancerous lesions, such as adenoma, serrated polyps, and dysplasia, must be confirmed before applying this technology in clinical endoscopic examination [15].

Indocyanine green (ICG) is a tricarbocyanine iodide dye molecule that is amphiphilic, relatively nontoxic fluorescent contrast agent exhibiting a good safety profile for hepatic function examinations [16, 17] and has been approved for clinical applications in the USA and in some European and Asian countries. It is known that intravenously injected ICG preferentially accumulated in malignant tissues than normal through its binding to macromolecular serum proteins or cellular endocytosis [18–22]. Based on the preferred chemical properties for *in vivo* imaging, various clinical and preclinical applications of ICG have been reported, including tumor detection in the liver [23, 24], breast [25], head and neck [26], lungs [27], ovaries [28], and colon [29, 30] of humans. Despite little background noise in the gastrointestinal tract under near infrared light exposure with ICG fluorescence endoscopy [31], verification of the practicality of ICG injections to detect colon cancer and precancerous lesions in the clinic setting has been insufficient.

Artificial intelligence (AI) has been widely applied in medicine, including colon cancer diagnosis and treatment. Recently, several authors have presented research on the application of AI in early diagnosis of colon cancer using convolutional neural networks (CNNs), a type of deep learning algorithm [32, 33]. In these studies, the possibility of the clinical application of AI was demonstrated by using white-light colon endoscopy, tomography (PET-CT), and histopathology images [34–36].

In the present study, we investigated the correlation between fluorescence intensity of ICG-based fluorescence endoscopy and colon cancer specific targets in a colitis-associated colon cancer model. To verify the feasibility of ICG for clinical endoscopy, we evaluated its ability to detect precancerous lesions by conducting AI analysis of ICG fluorescence endoscopy images. This successful trial of an AI integrated ICG fluorescence endoscopy image analysis demonstrated relevance in the interpretation of precancerous lesions of colon cancer patients. Furthermore, we evaluated the mechanism of ICG retention in tumor cells and its relation with inducible nitric oxide synthase (iNOS) expression using a cell culture experiment. Finally, we validated the correlation between fluorescence intensity and malignant lesions in patient tissues.

## Materials and methods

### Patient consent for human participants

All patient tissue samples and data used in this study were provided by the Institutional Review Board of Asan Medical Center (IRB; protocol No. 2017–0837). Comprehensive approvals for basic research were obtained from all patients, and informed consent was obtained by written from all patients and/or their legal guardian(s). This study was conducted in accordance with the Ethical Guidance of the Declaration of Helsinki.

### Animal study ethics

All animal studies were approved by Institutional Animal Care and Use Committee of the Asan Institute for Life Sciences, Asan Medical Center (IACUC; Approval No. 2019-12-205). All animal care and experimental procedures aligned with appropriate instructions and regulations of the Institutional Animal Care and Use Committee (IACUC) at Asan Medical Center and ARRIVE guidelines.

### Cell lines

The human colon cancer cell lines, HCT116, SW480, and CCD841 were obtained from the American Type Culture Collection (ATCC; Manassas, VA, USA). Cells were maintained in Dulbecco’s Modified Eagle Medium/High Glucose (DMEM/High Glucose; Gibco; Thermo Fisher Scientific, Inc., Waltham, MA, USA) supplemented with 10% fetal bovine serum (Hyclone, Logan, UT, USA) and 1% Antibiotic-Antimycotic (Gibco; Thermo Fisher Scientific, Inc.). The cell lines were grown at 37°C in a humidified incubator containing 5% CO_2_.

### Establishment of the azoxymethane/dextran sulfate sodium (AOM/DSS)-induced colitis-associated colon cancer in a mouse model

15 Male Balb/c mice (age, 6–7-week-old; weight, 20–23 g) were purchased from Orient Bio (Seongnam, South Korea). All animals were kept in polycarbonate cages (3 mice per cage) under controlled environmental conditions at 22°C and 50% humidity in a 12 hours light/dark cycle. Enough food and water were made available to the animals. Starting from 7 to 8 weeks old, mice were administered a single intraperitoneal injection of azoxymethane (AOM) (10 mg/kg body weight; Wako Pure Chemical Co., Osaka, Japan). One week after the AOM injection, colitis was induced by two intermittent one-week-cycle administration of 2% dextran sodium sulfate (DSS; MP Biochemicals, Santa Ana, CA, USA; MW 36,000–50,000) in drinking water. Mice that showed critical weight loss during the first week of DSS administration had their DSS treatment discontinued immediately. One week after discontinued DSS administration, tumor progression was confirmed weekly through optical/fluorescent colonoscopy (Vetcom; Karl Storz, Tuttlingen, Germany). 10~14 weeks after AOM administration, all mice were sacrificed by inhalation anesthesia (isoflurane; Terrell, Piramal Critical Care, PA, USA), excised, and opened longitudinally to remove their colons (S1 Fig in S1 File). Colons were cleaned with PBS and number of polyps from the start of cecum to the anus. Three sections (~2 cm) of rectum samples were recovered from each mouse, fixed in 4% formalin embedded in paraffin or flash-frozen in liquid nitrogen and stored at −80°C until analyzed for histology. All animals were kept in polycarbonate cages (3 mice per cage) under controlled environmental conditions at 22°C and 50% humidity in a 12 hours light/dark cycle. Enough food and water were made available to the animals. No animals were found dead and any mice were not reached endpoint criteria written in approved Animal Care and Use Protocols (IACUC).

### Orthotopic colonic submucosal implantation of CRC cells

Before the orthotopic submucosal injection was performed, a fabricated needle was prepared for the working channel of the colonoscopy (Vetcom; Karl Storz). A 30G needle was bound to a 23G needle with a flexible plastic pipe. Thereafter, 1 × 10^7^ HCT116 and SW620 cells in 50–100 μL of 10% Matrigel (Corning, NY, USA)/phosphate buffered saline (PBS) were injected into 8–10-week-old balb/c mice (Male, Orient Bio Inc.). A colonic submucosal injection was gently inserted into the working channel of the endoscope using the modified flexible needle. After injection, tumor formation was confirmed through colonoscopy imaging every week. Colonoscopy was performed using fluorescence endoscopy (Vetcom; Karl Storz).

### Real-time fluorescence endoscopy imaging of mouse colon tumor

ICG was purchased from USP (1340009, USP, Rockville, MD, USA), and was dissolved in distilled water with 5% glucose (Sigma-Aldrich Co., St. Louis, MO, USA). All colonoscopies were performed using the animal endoscopic system (Vetcom; Karl Storz). The IMAGE1 H3-ZF1 THREE-CHIP FULL HD Camera system with fluorescent filters (wavelengths 805 nm–835 nm) that enable ICG fluorescence detection in the basic endoscope and the modified D-light PVET source (66100M3) were added. All endoscopy images were recorded as mp4-video files using the AIDA HD control system (Karl Storz) and sequences of endoscopy images were converted into TIF images through snapshots. The endoscopy image fluorescence intensity was analyzed by ImageJ (NIH, Bethesda, MD, USA). Regions of interest (ROIs) were defined around the detected polyp based on the corresponding optical images. Thereafter, ROIs of polyps were compared to adjacent normal mucosa and documented as a tumor-to-normal ratio for each site. After tumor implantation, orthotopic mice were administered ICG via tail vein injection. Thirty minutes later, endoscopy was performed and images were obtained with the animal endoscopic system (Vetcom; Karl Storz). To confirm the intensity of fluorescence detection in *ex vivo*, molecular imaging was performed using the Xenogen *in vivo* imaging system (IVIS) spectrum system (Caliper Life Sciences, Inc., Hopkinton, MA, USA).

### Ex vivo molecular imaging of CRC patients

Human colon tissue samples (10‒20 mm) were surgically excised from eight patients. Each fresh CRC tissue with adjacent normal colon tissue was evaluated immediately following surgery. Autofluorescence images of the tumor tissues and paired normal colon tissues were obtained prior to ICG incubation. Before incubation, to prevent a possible false-positive effect from probe infiltration of the resection, the tissue was covered with low-melting agarose gel. CRC tissues and paired normal colon tissues were incubated with ICG (5 mg/mL) for 30 min. After incubation, tissues were washed three times with PBS, and fluorescence molecular imaging was performed with the Xenogen IVIS spectrum system (Optix MX3 system; ART Advanced Research Technologies Inc., Montreal, Canada).

### Histology and immunohistochemistry

Immunohistochemistry (IHC) was performed using patient colon tissues, which were fixed with 4% paraformaldehyde and embedded in paraffin. Immunostaining was performed using the BenchMark XT automatic immunostaining device (Ventana Medical Systems, Inc., Oro Valley, AR, USA) and OptiView DAB IHC Detection (Ventana Medical Systems, Inc.). Tissue sections (4 μm) were transferred to salinized, charged slides and incubated at room temperature and 65°C. After antigen retrieval for 64 min, the sections were incubated on a fully automated immunostainer with anti-iNOS antibody (Abcam, Cambridge, UK;) for 32 min. Tissue section slides were then counter-stained with 4′,6-diamidino-2-phenylindole (DAPI). All image-staining patterns of the slides were acquired using an OptiView DAB IHC Detection Kit (Ventana Medical Systems, Inc.). Furthermore, all images were acquired using a x20 objective lens.

### Immunofluorescence

Tissue cryosections fixed with 4% paraformaldehyde were incubated with iNOS antibody, followed by incubation with anti-rabbit IgG Alexa Fluor ^TM^ 488 (Thermo Fisher Scientific). Microscopic images were obtained by using ZEISS Axio Observer (Zeiss, Oberkochen, Germany).

### Detection of time-based ICG accumulation in vitro

The tested cell lines (HCT116, SW480, and CCD841) were seeded in 96-well plates at a density of 1 × 104 cells per well in DMEM/ High Glucose medium with 10% FBS. The cells were washed with serum-free media and incubated with ICG (50 μM) in 1% FBS media for various durations (0, 1, 5, 10, 20, 30, and 60 min and 12, 18, and 24 h) at 37°C. After incubation, the cells were washed three times with PBS. To confirm the intensity of ICG accumulation in vitro, fluorescence molecular imaging was performed using the Xenogen IVIS spectrum system (Caliper Life Sciences, Inc.MA, USA).

### Western blot analysis

The cell lysates were prepared using radioimmunoprecipitation assay (RIPA) lysis buffer (Thermo Fisher Scientific, Inc.) and protease inhibitor cocktail (GenDEPOT, Barker, TX, USA). Cell lysates were separated using 10% sodium dodecyl sulphate-polyacrylamide gel electrophoresis (SDS-PAGE) and transferred to polyvinylidene fluoride (PVDF) membranes. Protein-transferred membranes were blocked with 5% bovine serum albumin (BSA) in Tris-buffered saline Tween 20 (TBS-T) for 1 h at room temperature and incubated with blocking buffer-diluted primary antibodies, including E-cadherin (Abcam; ab76055), iNOS (Abcam; ab178945), c-Met (Abcam; ab51067), VEGFA (Abcam; ab46154), Ki-67 (Abcam; ab16667) and ABCB1 (Abcam; ab170904) antibody (1:1000) at 4°C overnight. After the membranes were washed and incubated with secondary antibodies, immunoreactive protein expression signals were detected using the enhanced chemiluminescence (ECL) substrate (Thermo Fisher Scientific, Inc.) and visualized using Luminograph III (ATTO Corporation, Tokyo, Japan). The expression of anti-β-actin (Sigma-Aldrich Co.) was used as the loading control.

### Intracellular nitric oxide assay

The tested cell lines (HCT116, SW480, and CCD841) were seeded in 96-well plates at a density of 1 × 10^4^ cells per well. Quantification of nitric oxide synthase (NOS) activity in a 96-well format was performed using the manufacturer’s instruction. Briefly, the nitric oxide fluorometric probe in working solution was added to each well and incubated at 37°C in the dark for 2 h. Cell lysis buffer was then added to each well without washing before taking the measurement. The fluorescence intensity at excitation/emission wavelengths of 480/530 nm was measured using a CLARIOstar microplate reader (BMG Labtech, Ortenberg, Hessen, Germany).

### Artificial intelligence analysis of fluorescence endoscopy images

Sixty images (optical, fluorescence) obtained during endoscopic observations of the AOM/DSS mouse model, together with the deep learning network, were used to categorize four classes of CRC development. First, data preprocessing was performed before inputting it into the network. For data preprocessing, 16 types of augmentation were performed to extract specific features that could improve the accuracy of the network. We aimed for a robust network with high accuracy by adjusting the brightness of the dark image, emphasizing characteristics of the fluorescently marked part, and changing the image shape in various ways. EfficientNet [37] was used as a deep learning network, and a baseline architecture with an input resolution of 224 × 224 was applied. Performance of the network was evaluated using precision, recall, f1-score, and accuracy values. Parameters for network training were 300 epochs, the optimizer was AdamW, and the learning rate was 5.0e-4.

### Statistical analyses

Data are presented as mean ± standard error of the mean (SEM). Significant differences were evaluated using a paired t-test. Correlation analysis and coefficient calculation were performed with GraphPad Prism (GraphPad Software, CA, USA).

## Results

### Correlation analysis of fluorescence intensity and molecular targets for ICG fluorescence endoscopy imaging

**Fig 1:**
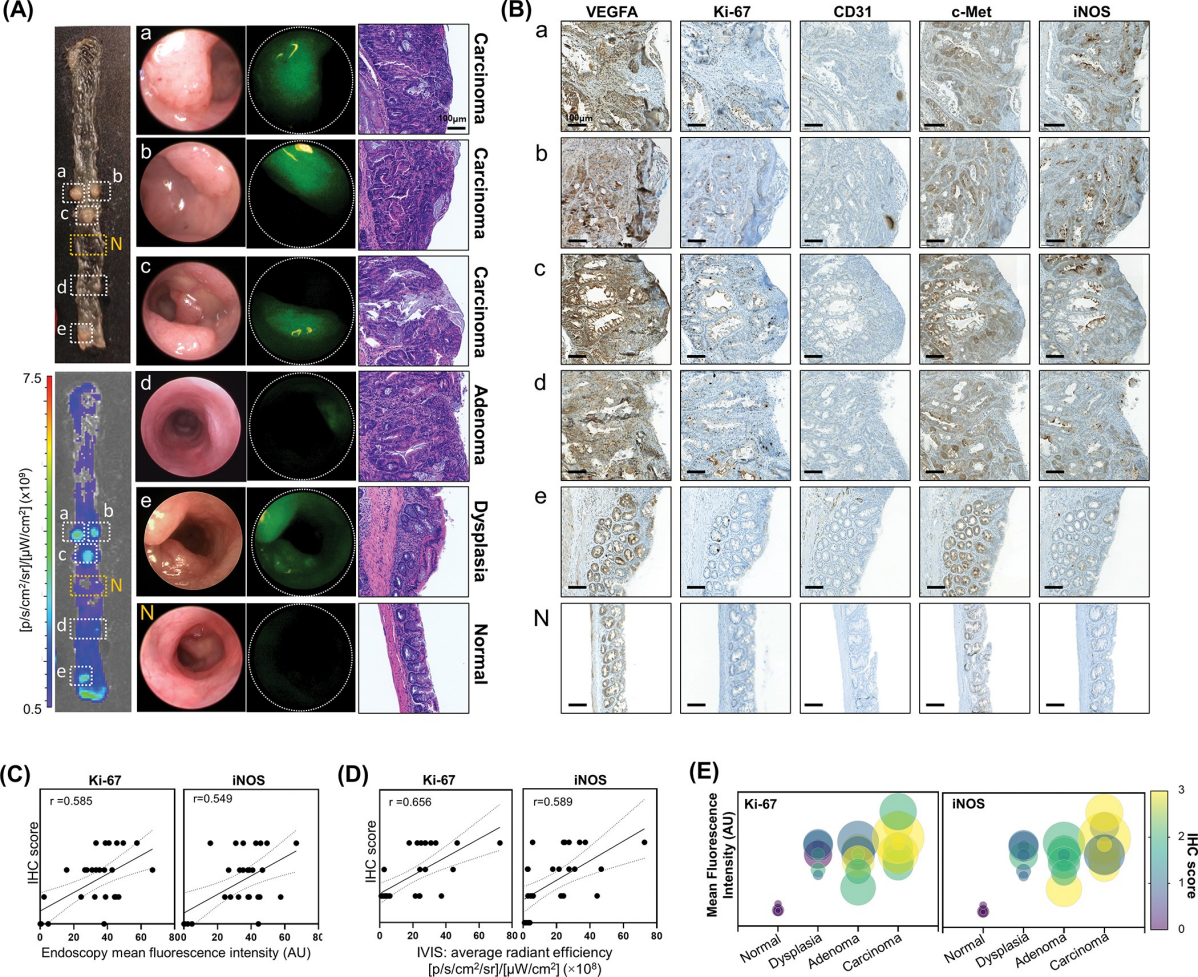
Correlation analysis of ICG fluorescence intensity and molecular targets for tumor imaging. (**A**) Representative indocyanine green (ICG) fluorescence endoscopic images and *ex-vivo* molecular imaging of polyps and adjacent normal lesions from a mouse colon with the corresponding hematoxylin and eosin-stained images (magnification, x200). (**B**) Immunohistochemistry (IHC) images with VEGFA, Ki-67, CD31, inducible nitric oxide synthase (iNOS), and c-Met for the corresponding lesions (**A**) (Scale bar: 100 μm). (**C**) Correlation analysis between endoscopy fluorescence intensity value (AU) of ICG accumulation and the IHC score of Ki-67 and iNOS. (**D**) Correlation analysis between *ex vivo* molecular imaging fluorescence intensity values of ICG and the IHC score of Ki-67 and iNOS. (**E**) Correlation analysis between ICG intensity values and the IHC score of Ki-67 and iNOS based on tumor stages. Data are presented as mean and error (95% confidence interval [CI]). Size of circle, average radiant efficiency from *ex vivo* molecular images ([p/s/cm^2^/sr]/[μW/cm^2^] (×10^8^)).

Colitis-associated colon cancer was induced in 14 mice using AOM/DSS. After a second injection of DSS, all mice were monitored every week with endoscopy, and proliferative lesions of various sizes and phases were observed (S1 Fig in S1 File). At monitoring week 5–6, all mice underwent a colon endoscopy, which revealed multiple tumor polyps that were identified as dysplasia, adenoma, and carcinoma via histological examinations (S2A Fig in S1 File). ICG was used to help visualize and interpret precancerous lesions. After 30 minutes from the ICG injection, the fluorescence signal of most polyps was shown to increase while maintaining a low background, whereas the colon of a normal mouse showed weak signals in both real time fluorescence endoscopy and *ex vivo* molecular imaging (Fig 1A, S3 Fig in S1 File). To further examine the ICG molecular imaging of tumor growth, the fluorescence intensity value of ROI of 25 tumor lesions and their adjacent normal lesions was quantified based on real time fluorescence endoscopy and *ex vivo* molecular imaging. Various ICG fluorescence intensity values were detected for most polyps, which significantly increased compared to those of normal lesions (S2B, S2C Fig in S1 File). For the ICG fluorescence intensity values, the average ICG signals of dysplasia, adenoma, and carcinoma were 1.8-fold, 2.8-fold, and 3.1-fold higher than that of the normal lesion, respectively (Table 1). Cases with 1.5-fold higher intensity than the normal lesions were analyzed via real time fluorescence endoscopy; all carcinoma lesions exhibited over 2-fold higher intensity. Furthermore, 90% (9/10 cases) of adenoma and 57% (4/7 cases) of dysplasia were found to have more than 1.5-fold higher fluorescence intensity than that of the adjacent normal lesion via real time fluorescence endoscopy (Table 1, S2 Fig in S1 File).

**Table 1:** Visualization percentages of precancerous lesions using indocyanine green (ICG) fluorescence endoscopy.

	Dysplasia	Adenoma	Carcinoma
Tumor/Normal	1.76 ±0.71	2.84 ±1.78	3.10 ±1.31
% cases > 1.5-fold	57 (4/7)	90 (9/10)	100 (9/9)

To further analyze tumor status and ICG fluorescence intensity, we calculated the correlation of fluorescence intensity values, histology, and tumor specific molecules used for molecular imaging of colon cancer. A total of 25 polyps and five samples of normal tissue from 14 AOM/DSS mice were examined for correlation analysis (Fig 1A, S4 Fig in S1 File). IHC of proliferative, cancer specific, vascular targeting, inflammatory molecules, including c-Met, Ki-67, VEGFA, cluster of differentiation 31 (CD31), and iNOS, were scored based on an inspection by a pathologist and relevant references (Fig 1B, S4 Fig in S1 File) [17, 38, 39]. As shown in Fig 1C and 1D, the ICG fluorescence intensity was positively correlated with Ki-67 and iNOS. The correlation coefficient between CD31 and ICG intensity of real time fluorescence endoscopy was 0.447, whereas that with *ex vivo* molecular imaging was 0.144 (S7 Fig in S1 File). Moreover, results indicate that Ki-67 and iNOS had higher IHC scores for dysplasia than normal tissue, while the highest IHC scores were found for carcinoma, which is consistent with the ICG fluorescence intensity (Fig 1E).

### Cellular nitric oxide concentration is related to the preferential accumulation of ICG in tumors

**Fig 2:**
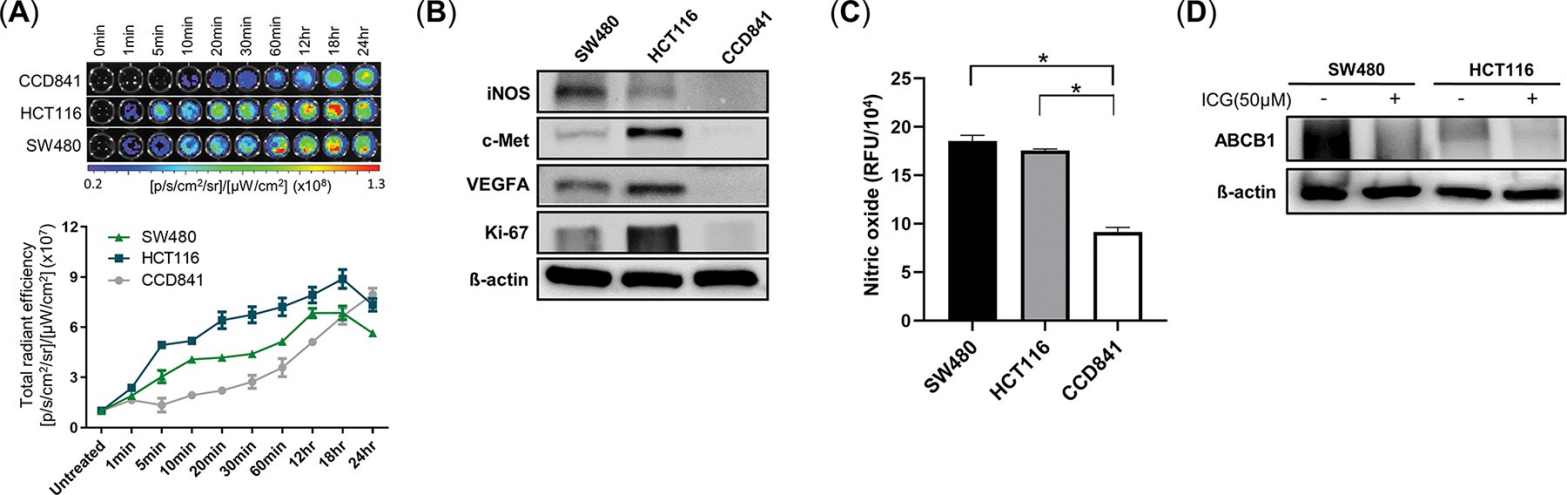
*In vitro* colon cancer cellular accumulation of ICG and iNOS/NO levels. **(A)** Time-based indocyanine green (ICG) accumulation in a normal cancer cell line (CCD841) and colon cancer cell lines (HCT116 and SW480). A total of 1 × 10^4^ cells/well were treated with 50 μM of ICG for 30 min prior to *in vivo* imaging system (IVIS) imaging (n = 4) (**B**) Western blot analyses of VEGFA, Ki-67, inducible nitric oxide synthase (iNOS), and c-Met in SW480, HCT116, and CCD841. (**C**) Intracellular nitric oxide (NO) assessment in SW480, HCT116, and CCD841 cells. Values are expressed as mean ± standard error of the mean (SEM; *p < 0.05) (**D**) Western blot analyses of ABCB1 in SW480 and HCT116 with treatment of ICG for 30 min.

To elucidate the mechanism of preferential retention of ICG in malignant lesions, we examined ICG accumulation in cultured cells depending on incubation time. As shown in Fig 2A, the ICG signal detected in SW480 and HCT116 was significantly stronger than that in the normal CCD841 colon epithelial cell line up until 18 h of treatment. Further, there was no change in cell viability regardless of duration of ICG treatment (S5 Fig in S1 File). The cellular expression of Ki-67, VEGFA, c-Met, and iNOS also increased in SW480 and HCT116, whereas no expression was found in CCD841 (Fig 2B). Cellular nitric oxide is related to the inhibition of drug efflux [40]. After ICG treatment for 30 min, the intracellular concentrations of nitric oxide in SW480 and HCT116 were significantly higher than that in CCD841 (*p* < 0.0005, Fig 2C). To verify whether the efflux protein ABCB1 (p-gp) was inhibited, change in expression was investigated by immunoblotting, as shown in Fig 2D. Results demonstrate that ABCB1 was down-regulated after treatment with ICG for 30 min in cancer cell lines.

### Correlation of ICG molecular imaging and iNOS expression in CRC patients

**Fig 3:**
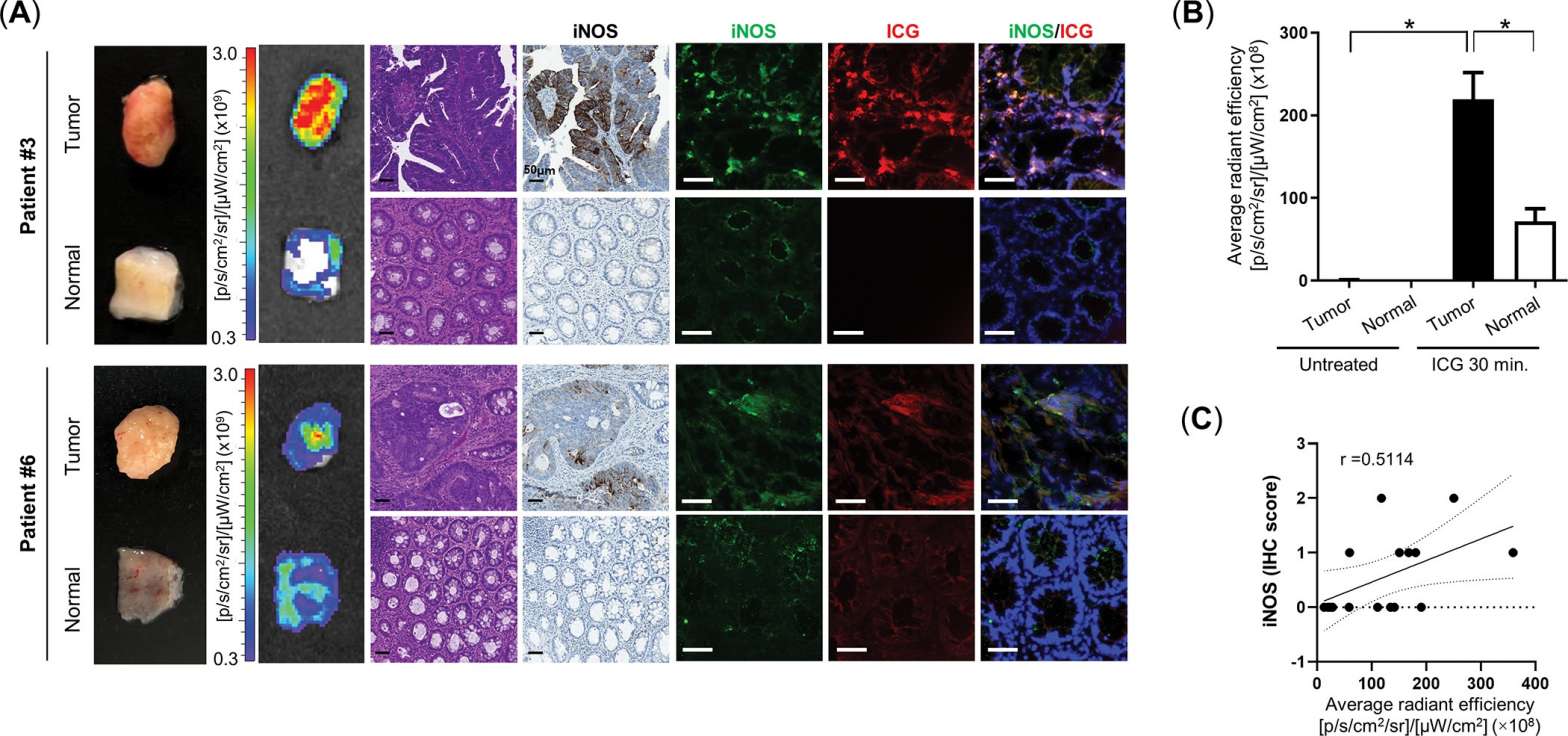
*Ex vivo* molecular imaging of tumor tissues from colorectal cancer (CRC) patients. **(A)** Representative *ex vivo* IVIS, hematoxylin and eosin, immunohistochemistry (IHC), and immunofluorescence images of CRC patient tissues with strong (upper panel) and weak (bottom panel) intensity of indocyanine green (ICG). (Scale bar: 50 μm) (**B**) Average fluorescence intensity of CRC patient specimens treated with ICG (*p < 0.05). (**C**) Correlation analysis between ICG fluorescence intensity value (average radiant efficiency) and the IHC score of inducible nitric oxide synthase (iNOS) expression in CRC patient tissues.

We analyzed the correlation between ICG fluorescence intensity and expression levels of both iNOS and c-Met using 16 samples from eight patients with CRC (Table 2) via *ex vivo* molecular imaging. After 30 min from ICG treatment, the fluorescence intensity and iNOS expression were measured in tumor and normal tissues (Fig 3A, S6 Fig in S1 File). As shown in Fig 3B, the ICG fluorescence signals of tumor tissues were significantly higher than those of normal tissues (*p* = 0.0003). Furthermore, the iNOS expression of tumor tissues was increased compared to that of normal tissues and positively correlated with the ICG fluorescence intensity (r = 0.5114, Fig 3C). ICG fluorescence signal and iNOS expression were observed in the same location (Fig 3A). c-Met expression in the tumor was increased as described previously and had a high positive correlation with ICG fluorescence intensity (S7 Fig in S1 File). To assess the effectiveness of ICG for detecting tumor lesions with increased iNOS expression *in vivo*, orthotopic tumor-bearing mice were evaluated via real time fluorescence endoscopy. Mice with confirmed tumor growth were administered ICG (7.5 mg/kg body weight) via the tail vein. After 30 min, the accumulation of ICG in tumor lesions with minimal autofluorescence background was detected and maintained for 1 h (S8A Fig in S1 File). The expression levels of iNOS and c-Met were detected using IHC staining, which revealed increased expression in tumor with high ICG fluorescence signals (S8B Fig in S1 File).

**Table 2:** Clinical information of colorectal cancer patients.

Characteristics	No. of patients	%
**Sex**		
Male	4	50
Female	4	50
**Age (Yr)**		
≥55	5	63
<55	3	37
**Histology**		
Adenoma	0	0
Well differentiated adenocarcinoma	0	0
Moderate differentiated adenocarcinoma	8	100
Poor differentiated adenocarcinoma	0	0
**Lymph node metastasis**		
Absent	1	12
Present	7	88

### AI-integrated ICG-fluorescence endoscopy analysis for detection of precancerous lesions in colon cancer development

**Fig 4:**
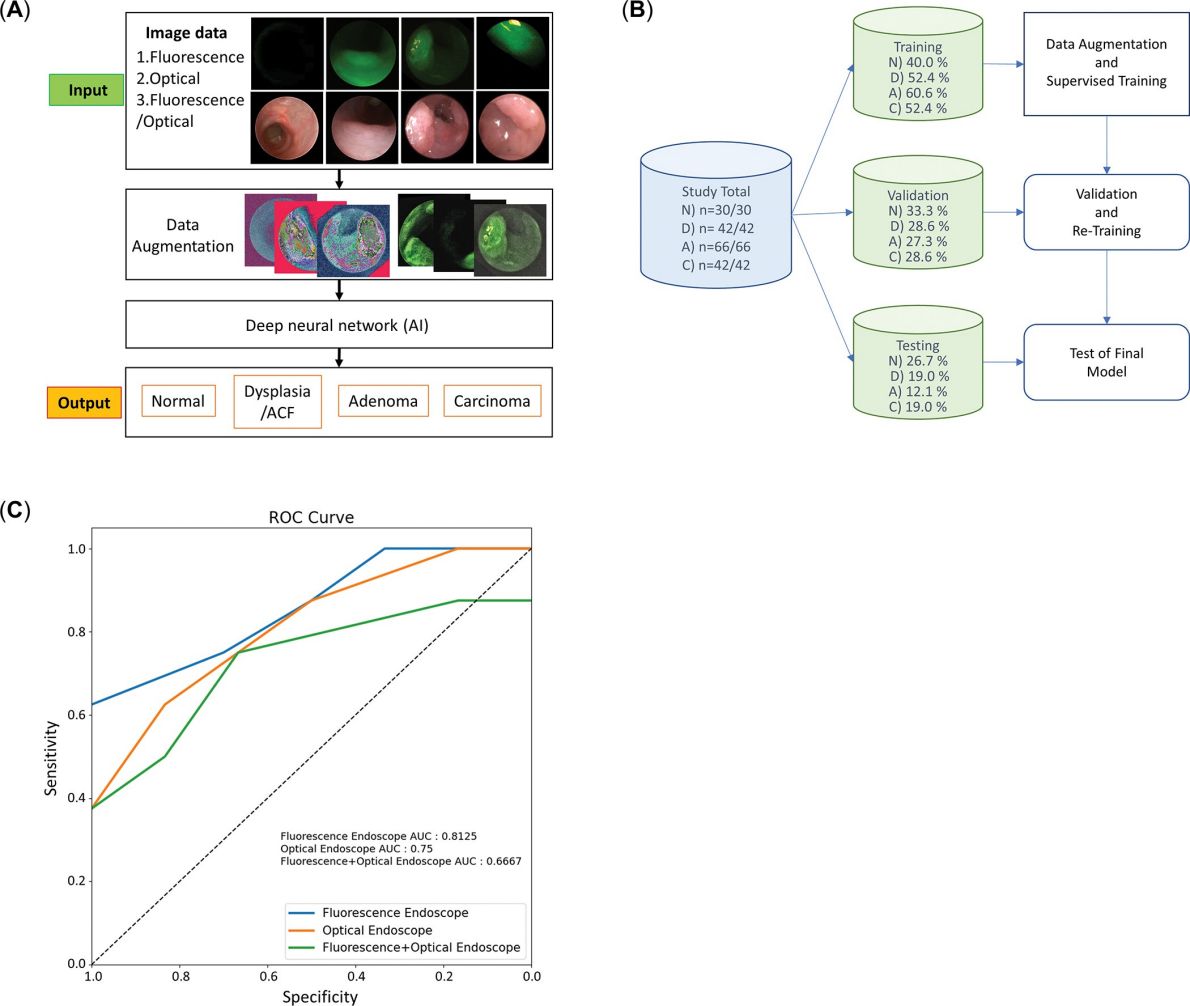
Deep learning network analysis of colon cancer fluorescence endoscopy. **(A)** Flow chart of image data and study design. **(B)** Schematic of the training/validation/test process used in this study. **(C)** Receiver operating curves of the artificial intelligence (AI) classification of 3 groups of images: fluorescence endoscopy, optical endoscopy, and fluorescence/optical images.

A total of 60 images (optical, fluorescence) together with histological diagnosis results from the endoscopic observations of the AOM/DSS mouse model were utilized for deep learning network analysis to categorize precancerous lesions into four classes; normal (N), dysplasia (D), adenoma (A), and carcinoma (C; Fig 4A, S4 Fig in S1 File). To prevent overfitting when training the AI on a small number of data, we performed data augmentation including transformations in scale, rotation, luminance/contrast, aspect ratio, and shearing among others (S9 Fig in S1 File). Training was performed with a 40%–60% of the total data set, followed by validation, which was performed with 27%–33% of the total data set (Fig 4B). Classification testing was performed using fluorescence, optical and fluorescence/optical images. Table 3 shows the accuracy of the classification of images into four classes using each image data set; this approach revealed that fluorescence images obtained the highest accuracy (0.750) for class predication of AI. Furthermore, we examined receiver operating characteristic (ROC) curves for three sets of images to assess the performance. As shown in Fig 4C, fluorescence endoscopy images yielded an area under the ROC curve (AUC) of 0.8125, whereas optical and fluorescence/optical images yielded 0.75 and 0.6667, respectively.

**Table 3:** Accuracy of the artificial intelligence (AI)-integrated endoscopy using deep neural network.

	Images	Fluorescence	Optical	Fluorescence/Optical
Accuracy		0.750	0.375	0.625
Precision	Normal	0.500	0.000	1.000
	Dysplasia	0.000	0.000	0.200
	Adenoma	1.000	0.500	1.000
	Carcinoma	1.000	0.250	0.500
Recall	Normal	1.000	0.000	0.500
	Dysplasia	0.000	0.000	0.500
	Adenoma	1.000	1.000	0.500
	Carcinoma	1.000	0.500	1.000
F1-score	Normal	0.667	0.000	0.667
	Dysplasia	0.000	0.000	0.500
	Adenoma	1.000	0.667	0.667
	Carcinoma	1.000	0.333	0.667

## Discussion

The preferential accumulation of ICG in tumor has been reported for various tumor types, including colon cancer [18, 21, 26, 41]. However, there are no reports on the potential of ICG-based real time fluorescence endoscopy imaging for detection of precancerous lesions in a clinical setting. In this study, we investigated the visualization ability of ICG endoscopy in colitis-associated colon cancer using an animal model and patient tissue samples. We report a successful trial of AI-integrated diagnosis and classification of precancerous lesions using fluorescence endoscopy. All carcinoma lesions could be detected with an average of 3.10-fold higher intensity; furthermore, 90% of adenoma lesions were detected with more than 1.5-fold higher intensity. The ICG intensity clearly correlated with the expression levels of iNOS, leading to an increase in nitric oxide concentration in tumor cells, which was related with the down-regulation of ABCB1 expression.

Numerous approaches have been employed to detect CRC lesions using fluorescently labeled antibodies or small molecules binding tumor cell [15]. Fluorescence labeled antibodies against EGFR and VEGF showed high specificity for the target tumor; however, these antibodies could not overcome the disadvantages such as longer time to reaction after injection, and immunogenicity [9, 11]. For enhanced ability, small fluorescence labeled peptide targeting c-Met was developed [14] and tested in 15 patients via intravenous injection to observe malignant polyps; however, limitations such as background signal and a low number of cases to confirm safety were reported. In this study, we validated the clinical feasibility of ICG fluorescence endoscopy for tumor detection. ICG has been used in clinics for more than 40 years [42] and the safety of its use in the human body has been confirmed. According to some studies, ICG can be applied to detect colon tumors in a colitis-associated colon cancer animal model [17, 43]. In the present study, the fluorescence intensity of ICG via real time fluorescence endoscopy and *ex vivo* molecular imaging was quantified, and the correlation coefficient between these values and the tumor growth in 25 polyps from animal models and 16 human tissue samples was calculated. ICG could detect 100% of carcinoma, 90% of adenoma, and 57.1% of dysplasia within 30 min after injection.

Ki-67 labeling is generally used as a marker of proliferation for tumor grading [44, 45] and was found to have a positive correlation with ICG intensity (r = 0.585) in our real time fluorescence endoscopy experiment. The tumor stage of patient tissues with a high percentage of Ki-67 labeling was T4 cancer whereas that of others was T3, as described previously. However, the ICG intensity and Ki-67 labeling were not directly correlated in human patient tumor tissues (r = 0.025). As all patient samples were surgically resected CRC tissues, the ICG intensity could only distinguish between normal and carcinoma tissues rather than cancer grades. The ICG fluorescence intensity was relatively correlated with c-Met expression in animal models and patient tissue samples (r = 0.443 and r = 0.759, respectively). c-Met is considered a suitable biomarker for detection of early stages of cancer, including adenoma [14, 46]. Recently, cyanine dye (Cy5) labeled peptide probe has been tested for fluorescence endoscopy detection in 15 CRC patients, and was reported to successfully detect most adenomatous polyps as well as more than half of hyperplasias [14]. These results are consistent with our correlation data of ICG fluorescence intensity in animal models. The correlation coefficient between ICG fluorescence intensity and vascular targeting molecules, including VEGF (r = 0.250) and CD31 (r = 0.447), was relatively low, which suggests that the tumor selectivity of ICG is related to an increase in vascular permeability rather than an angiogenic effect. Vascular permeability is promoted by inflammatory mediators, such as iNOS [47], which was highly correlated with ICG intensity as per our correlation analysis data. Meanwhile, our data revealed that ICG intensity is highly correlated with iNOS expression in the colitis-associated colon cancer mouse model and tissue samples from CRC patients (r > 0.5). In fact, expression levels of iNOS were demonstrated to be associated with poor survival rates in several cancers [48–50]. In addition, iNOS was shown to be up-regulated in most chronic inflammatory diseases and cancers, including ulcerative colitis and colon cancer [51]. Tumor cellular iNOS contributes to the production of nitric oxide in the presence of nicotinamide adenine dinucleotide phosphate (NADPH) and oxygen. In recent studies, an increase in nitric oxide concentration was shown to mediate drug efflux inhibition by inactivating ATP binding cassette (ABC) transporters, such as ABCB1 (p-gp), ABCC1 (MRP1) and ABCG2 (BCRP) in tumor cells [52–54]. Although previous studies have demonstrated that preferential ICG uptake is due to extravasation and endocytosis [22], the mechanism of tumor selective retention is poorly understood. Based on the *in vitro* data presented in this present study, concentration of cellular ICG detected in cancer cell lines differed from that of normal colon epithelial cells according to the ICG incubation time. Moreover, both iNOS expression and intracellular nitric oxide concentration were higher in cancer cell lines. We also observed that the expression of ABCB1 was down-regulated in cancer cells after a 30-minute ICG treatment. These results suggest that increased cellular nitric oxide concentration caused differences in ICG efflux in cancer cells compared to that in normal cells, resulting in prolonged retention in the short term (24 h). Indeed, a prior report revealed that the increase in nitric oxide concentration and iNOS expression was associated with a marked reduction in the doxorubicin efflux rate in colon cancer cell lines [40]. Since we have observed differences within 24 hours in in vitro experiments and in colitis-associated colon cancer, it is plausible that alternative mechanisms may exist in various types of cancer. Meanwhile, further research is needed to better understand the role of ICG in imaging inflammatory conditions.

For the first time, we demonstrated that ICG-fluorescence endoscopic images together with AI analysis enable accurate classification of precancerous lesions into four tumor classes. The accuracy of this approach was evaluated by implementation of AI and endoscopic images of colon cancer obtained from the AOM/DSS mouse model which showed the highest AUC (0.8125) by utilizing only fluorescence endoscopy images, while use of optical endoscopy images yielded an AUC of 0.75. Notably, the AUC of the fluorescence/optical endoscopy concurrent analysis was considerably lower (0.6667). These results suggest that ICG positive cells reflect colitis-associated tumor specific characters with intracellular nitric oxide concentration and this feature together with AI training could be utilized to improve clinical diagnostics.

In this study, we demonstrated that ICG together with AI could be employed for carcinoma and adenoma imaging in colitis-associated colon cancer patients. However, this study has some limitations. Notably, a small sample number could limit the universality of the results. Thus, the feasibility of ICG in detecting precancerous lesions in a larger human cohort needs to be investigated.

## Conclusion

We showed fluorescence endoscopy images analyzed with AI could detect precancerous lesion of colon cancer with higher accuracy than optical endoscopy images. Furthermore, the positive correlation of ICG intensity and expression of iNOS in patient tumor tissues was demonstrated. We suggested the novel mechanism that intracellular iNOS concentration is related to ICG retention in cancer cells.

## Supporting information

S1 File (ZIP)

S1 Raw images (PDF)
