# Supplementary material for: Investigation of artificial intelligence integrated fluorescence endoscopy image analysis with indocyanine green for interpretation of precancerous lesions in colon cancer
Source: PLoS One. 2023 May 25;18(5):e0286189. doi: 10.1371/journal.pone.0286189 (PMC10212120; doi:10.1371/journal.pone.0286189)

Investigation of artificial intelligence integrated fluorescence endoscopy image analysis with indocyanine green for interpretation of precancerous lesions in colon cancer.

Jinhyeon Kim[1], Hajung Kim[2], Yong Sik Yoon[3], Chan Wook Kim[4], Seung-Mo Hong[1,5], Sungjee Kim[6], Doowon Choi[7], Jihyun Chun[5], Seung Wook Hong[1,3], Sung Wook Hwang[1,3], Sang Hyoung Park[3], Dong-Hoon Yang[3], Byong Duk Ye[1,3], Jeong-Sik Byeon[3], Suk-Kyun Yang[3], Sun Young Kim[8*], and Seung-Jae Myung[1,3,9*]

[1]*Digestive Diseases Research Center, University of Ulsan College of Medicine, Seoul, Republic of Korea*

[2]*Convergence Medicine Research Center, Asan Medical Center, Seoul, Republic of Korea*

[3]*Department of Gastroenterology, Asan Medical Center, University of Ulsan College of Medicine, Seoul, Republic of Korea*

[4]*Department of Colon and Rectal Surgery, Asan Medical Center, University of Ulsan College of Medicine, Seoul, Republic of Korea*

[5]*Department of Pathology, Asan Medical Center, University of Ulsan College of Medicine, Seoul, Republic of Korea*

[6]*Department of Chemistry and School of Interdisciplinary Bioscience and Bioengineering, Pohang University of Science & Technology, Pohang, Gyeongbuk, Republic of Korea*

[7]*School of Interdisciplinary Bioscience and Bioengineering, Pohang University of Science & Technology, Pohang, Gyeongbuk, Republic of Korea*

[8]*Asan Institute for Life Sciences, Asan Medical Center, University of Ulsan College of Medicine, Seoul, Republic of Korea*

[9]*Edis Biotech, Songpa-gu, Seoul, Republic of Korea*

Corresponding authors:

E-mail: enthalpy98@gmail.com; sjmyung@amc.seoul.kr

* These authors contributed equally to this work.

# Uncropped western blot images used in Figure 2 (B) and (D).

Figure 2 (B)

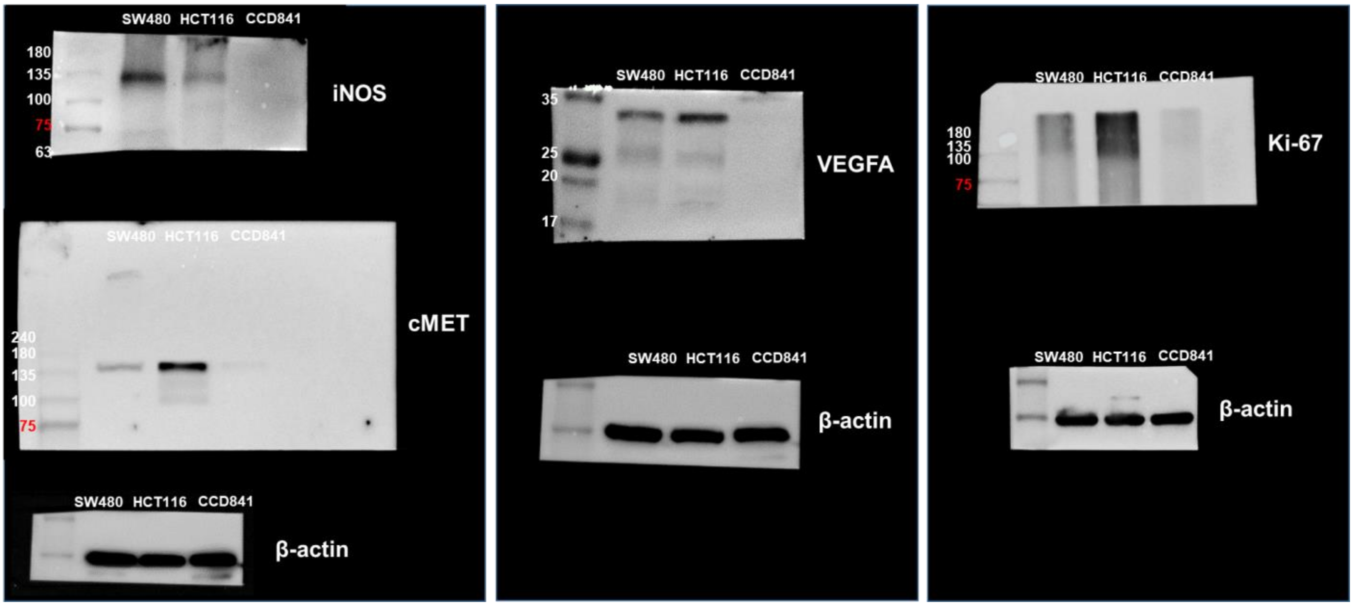

Figure 2 (D)

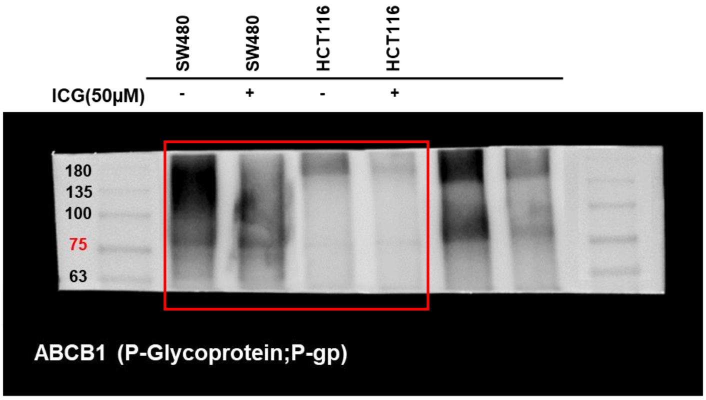

Datasheet (Observed band size: 180 kDa)
(Predicted band size: 141 kDa)

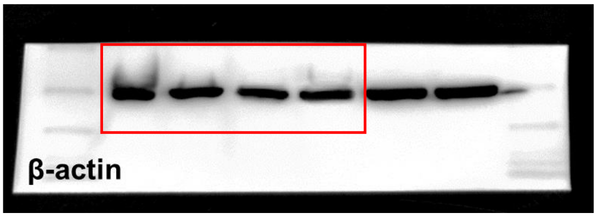

Supplement: S1 File — (ZIP) [file pone.0286189.s001.zip › Supporting information/S10 Fig_raw_images.pdf]
