# Supplementary material for: Investigation of artificial intelligence integrated fluorescence endoscopy image analysis with indocyanine green for interpretation of precancerous lesions in colon cancer
Source: PLoS One. 2023 May 25;18(5):e0286189. doi: 10.1371/journal.pone.0286189 (PMC10212120; doi:10.1371/journal.pone.0286189)
Supplement: S1 File — (ZIP) [file pone.0286189.s001.zip › Supporting information/S9 Fig.pdf]

Supplementary Figure S9.

	Parameter	Value
1	Additive-Gaussian-Noise	2.5500000000000003 to 12.75
2	GaussNoise	10.0 to 50.0
3	Normalize	mean = (0.485, 0.456, 0.406) std = (0.229, 0.224, 0.225)
4	Emboss	alpha : 0.2 to 0.5 / strength : 0.2 to 0.7
5	Perspective	scale : 0.05 to 0.1
6	Piecewise-Affine	scale : 0.03 to 0.05 nb_rows = 4 nb_cols = 4 interpolation = 1 keypoints_threshold : 0.01 to 0.5
7	CLAHE	tile_grid_size=(8,8), 1 to 4
8	Random-Brightness-Contrast	brightness_limit : -0.2 to 0.2 contrast_limit : -0.2 to 0.2
9	Random-Contrast	-0.2 to 0.2
10	RGB-Shift	r_shift_limit : -20 to 20 g_shift_limit : -20 to 20 b_shift_limit : -20 to 20
11	Rotate	-90° to 90°
12	HorizontalFlip	-
13	RandomRotate90	-
14	ShiftScaleRotate	shift_limit : -0.0625 to 0.0625 scale_limit : -0.1 to 0.1 ratate_limit : -45° to 45°
15	Affine	shear : -45 to 45
16	Resize	224x224x3
