# Supplementary figures and images for: Investigation of artificial intelligence integrated fluorescence endoscopy image analysis with indocyanine green for interpretation of precancerous lesions in colon cancer
Source: PLoS One. 2023 May 25;18(5):e0286189. doi: 10.1371/journal.pone.0286189 (PMC10212120; doi:10.1371/journal.pone.0286189)

Supplementary Figure S1.

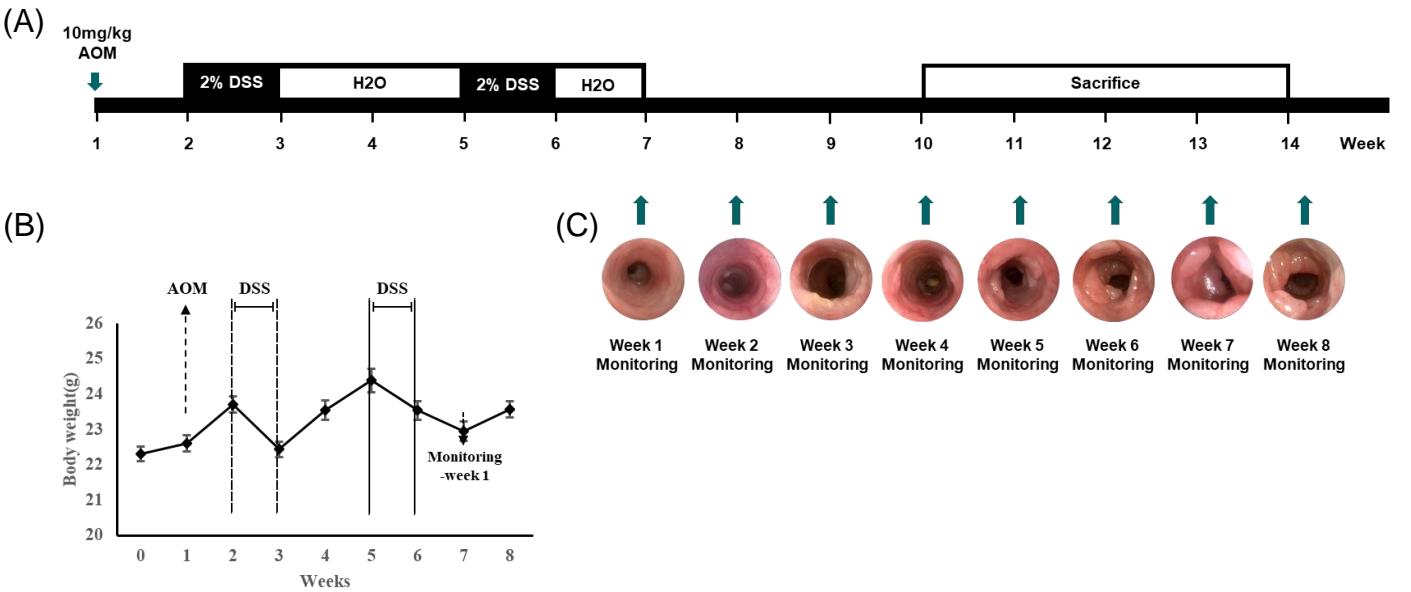

Supplement: S1 File — (ZIP) [file pone.0286189.s001.zip › Supporting information/S1 Fig.pdf]

Supplementary Figure S2.

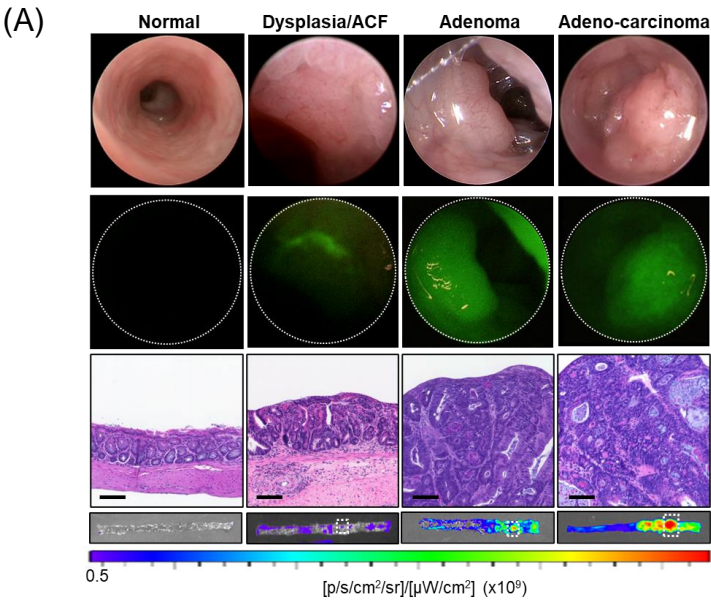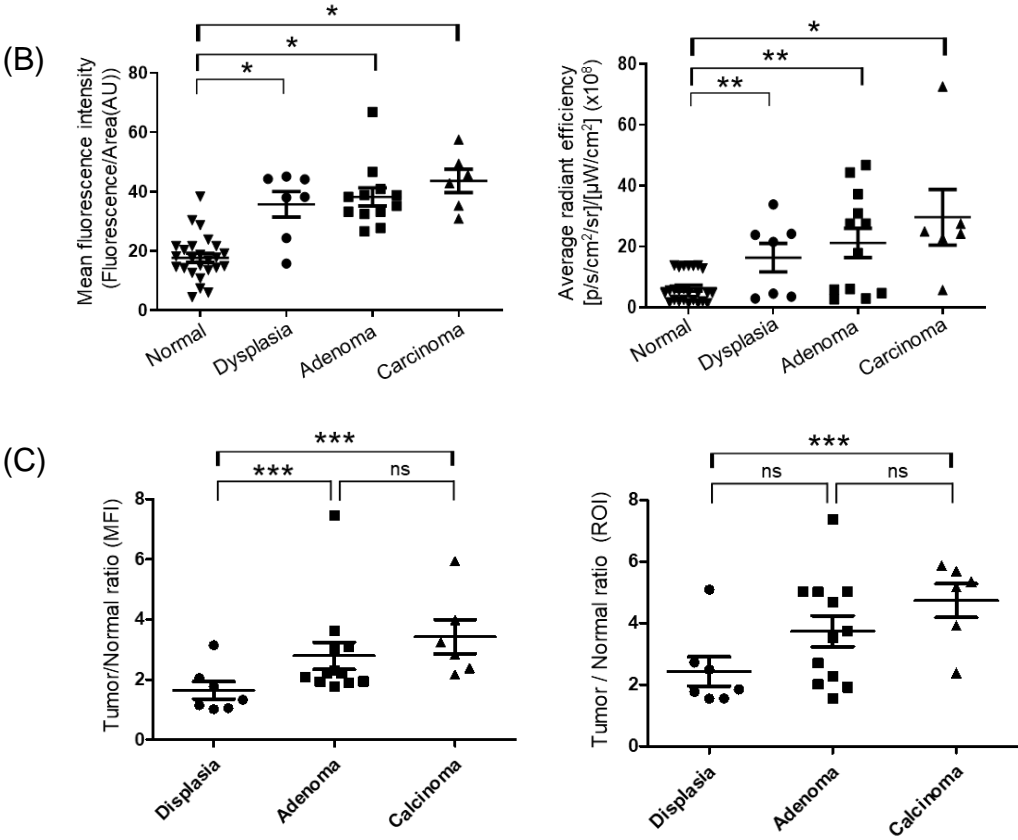

Supplement: S1 File — (ZIP) [file pone.0286189.s001.zip › Supporting information/S2 Fig.pdf]

Supplementary Figure S3.

(A)

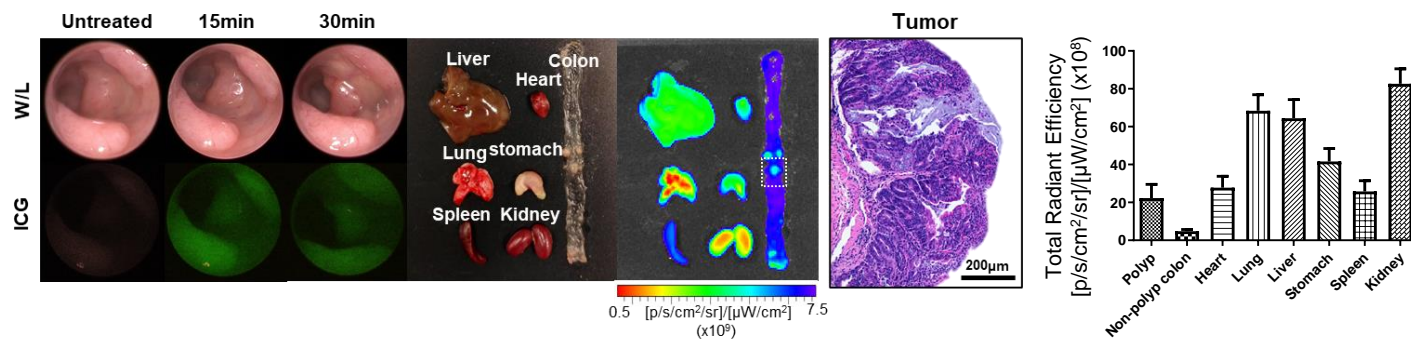

(B)

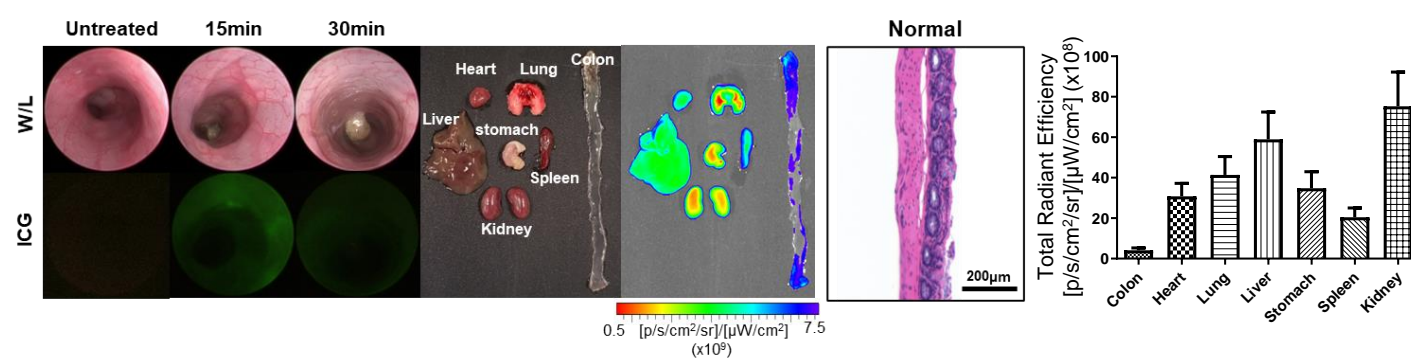

Supplement: S1 File — (ZIP) [file pone.0286189.s001.zip › Supporting information/S3 Fig.pdf]

Supplementary Figure S4.

(A)

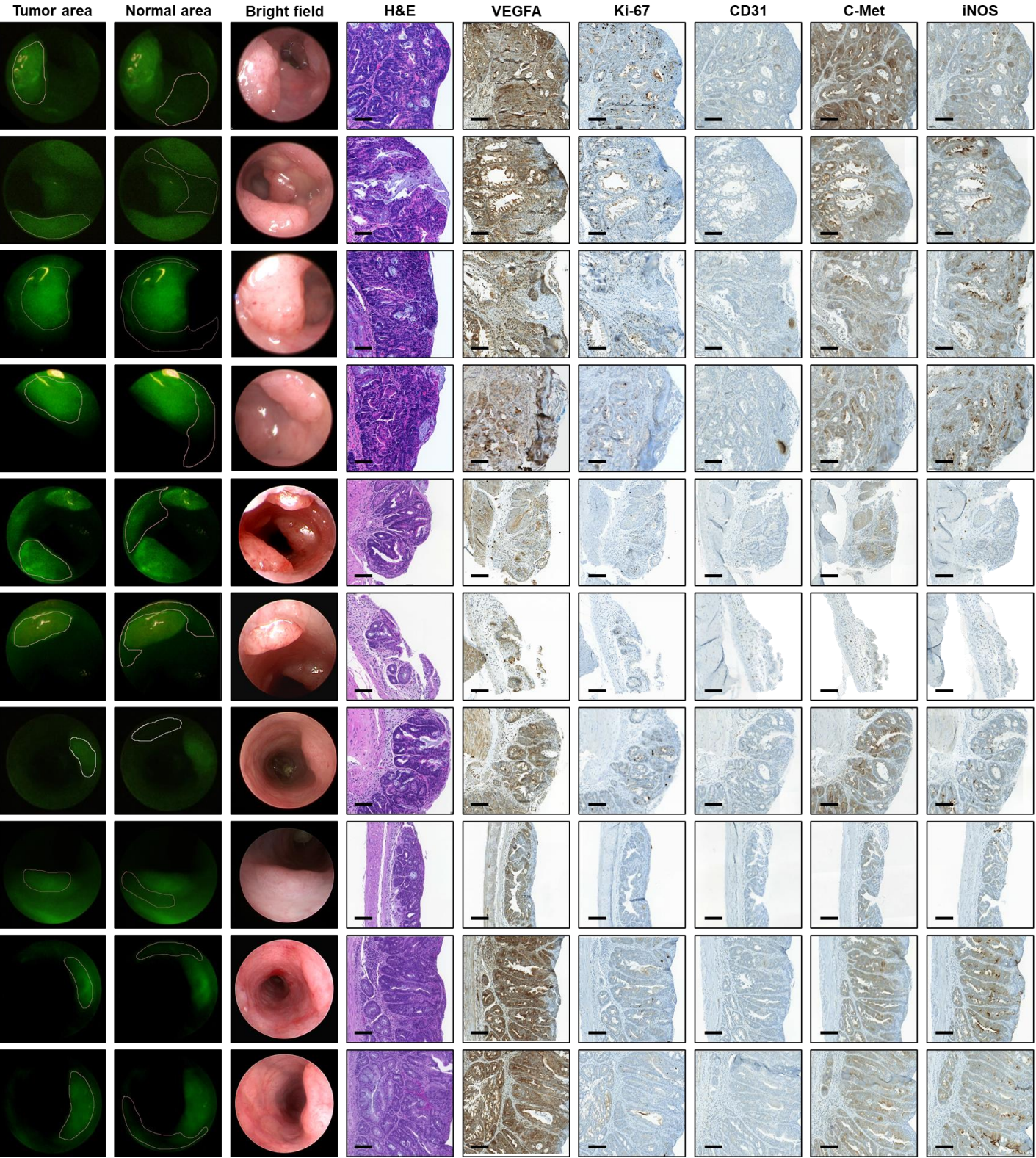

(A) Continued

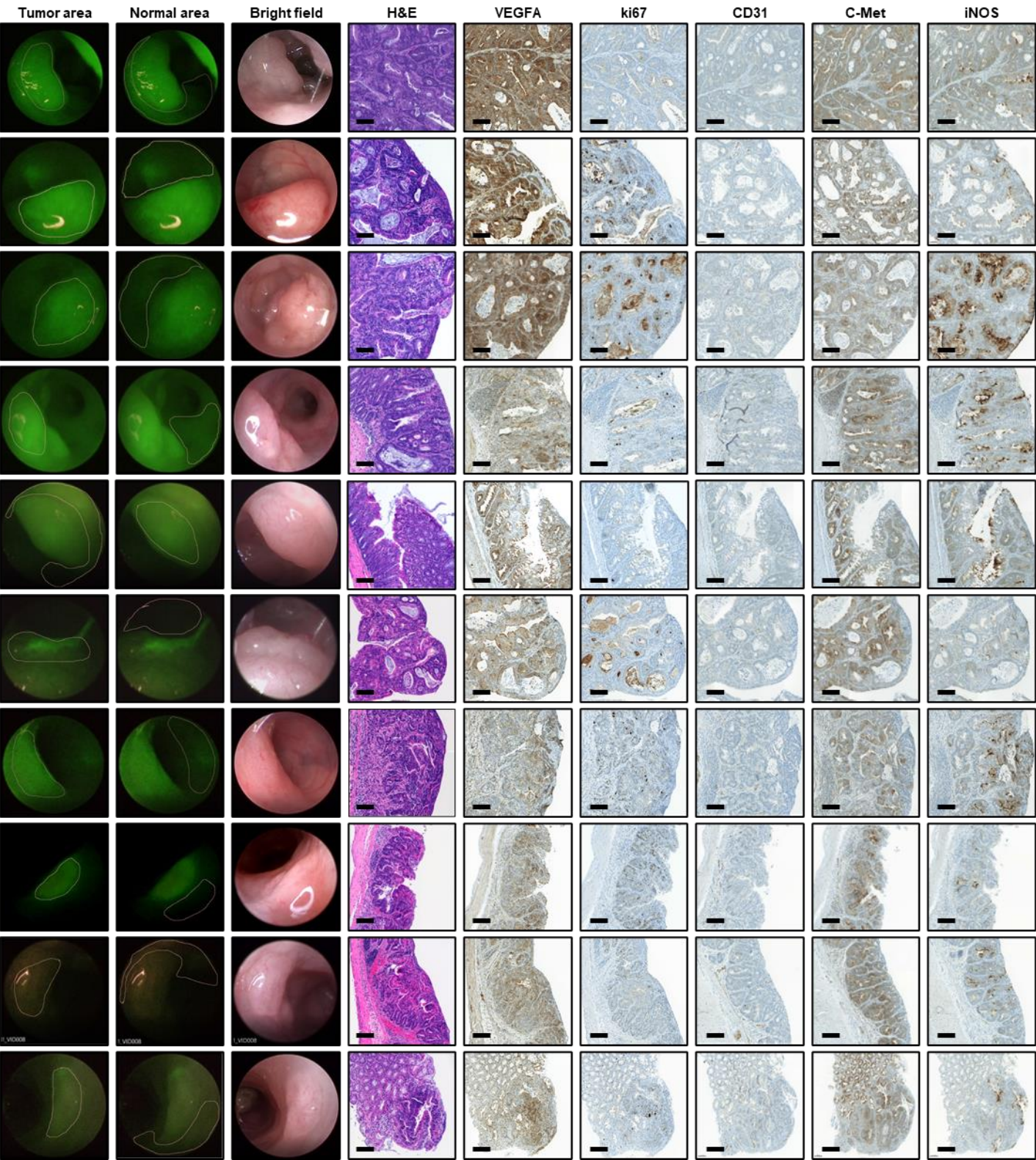

(A) Continued

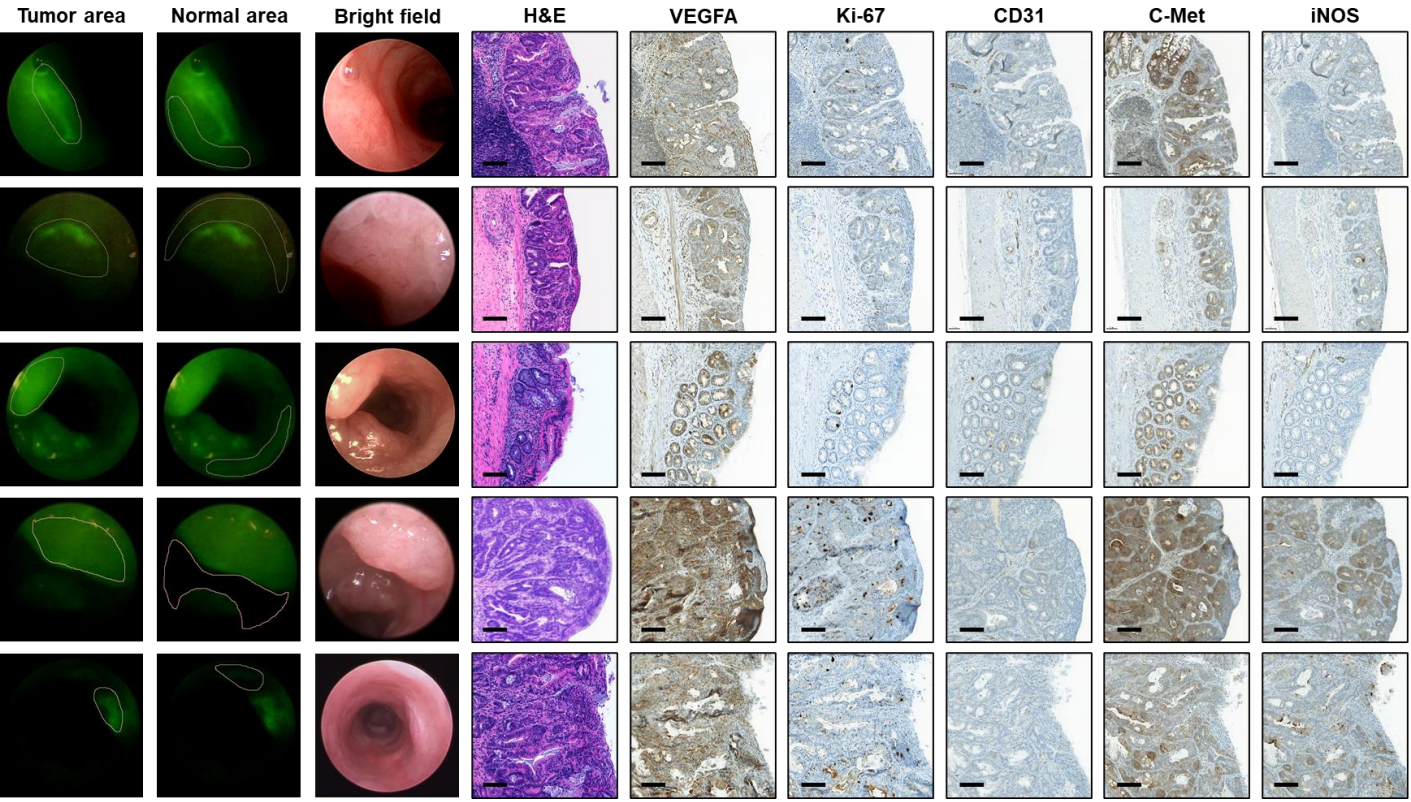

(B)

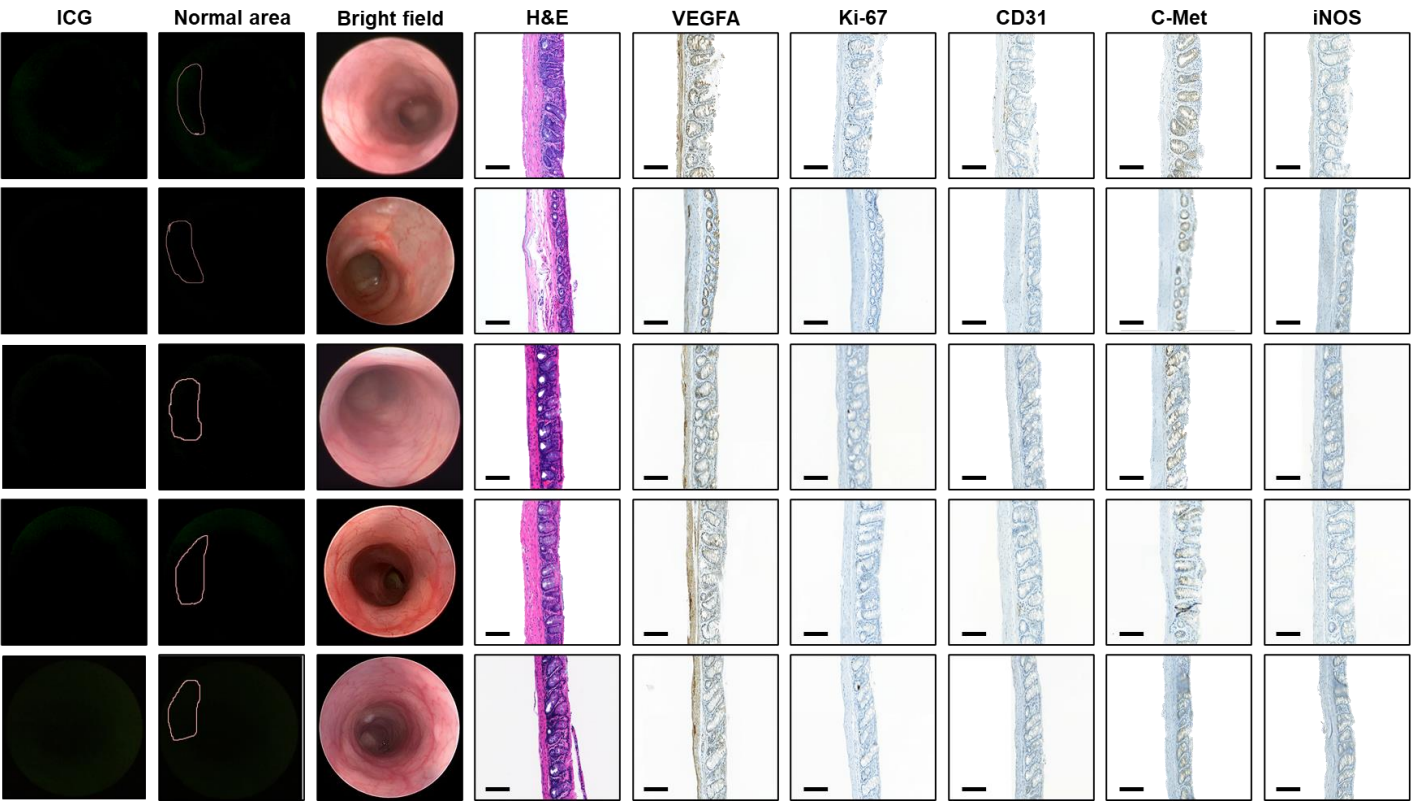

Supplement: S1 File — (ZIP) [file pone.0286189.s001.zip › Supporting information/S4 Fig.pdf]

Supplementary Figure S5.

(A)

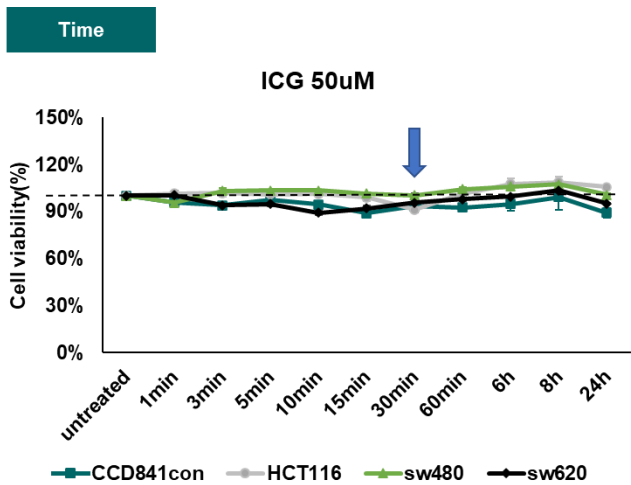

(B)

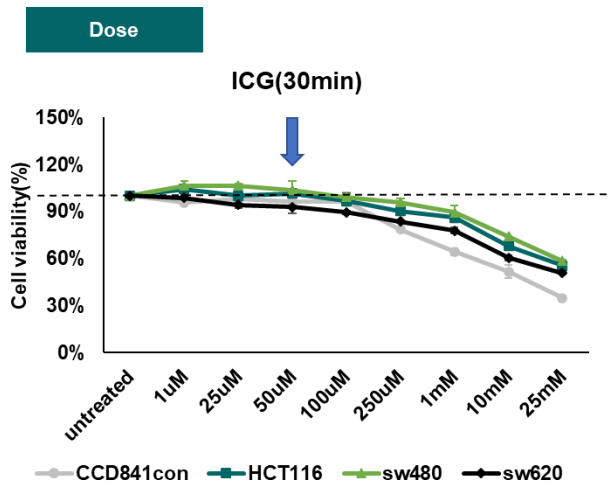

Supplement: S1 File — (ZIP) [file pone.0286189.s001.zip › Supporting information/S5 Fig.pdf]

Supplementary Figure S6.

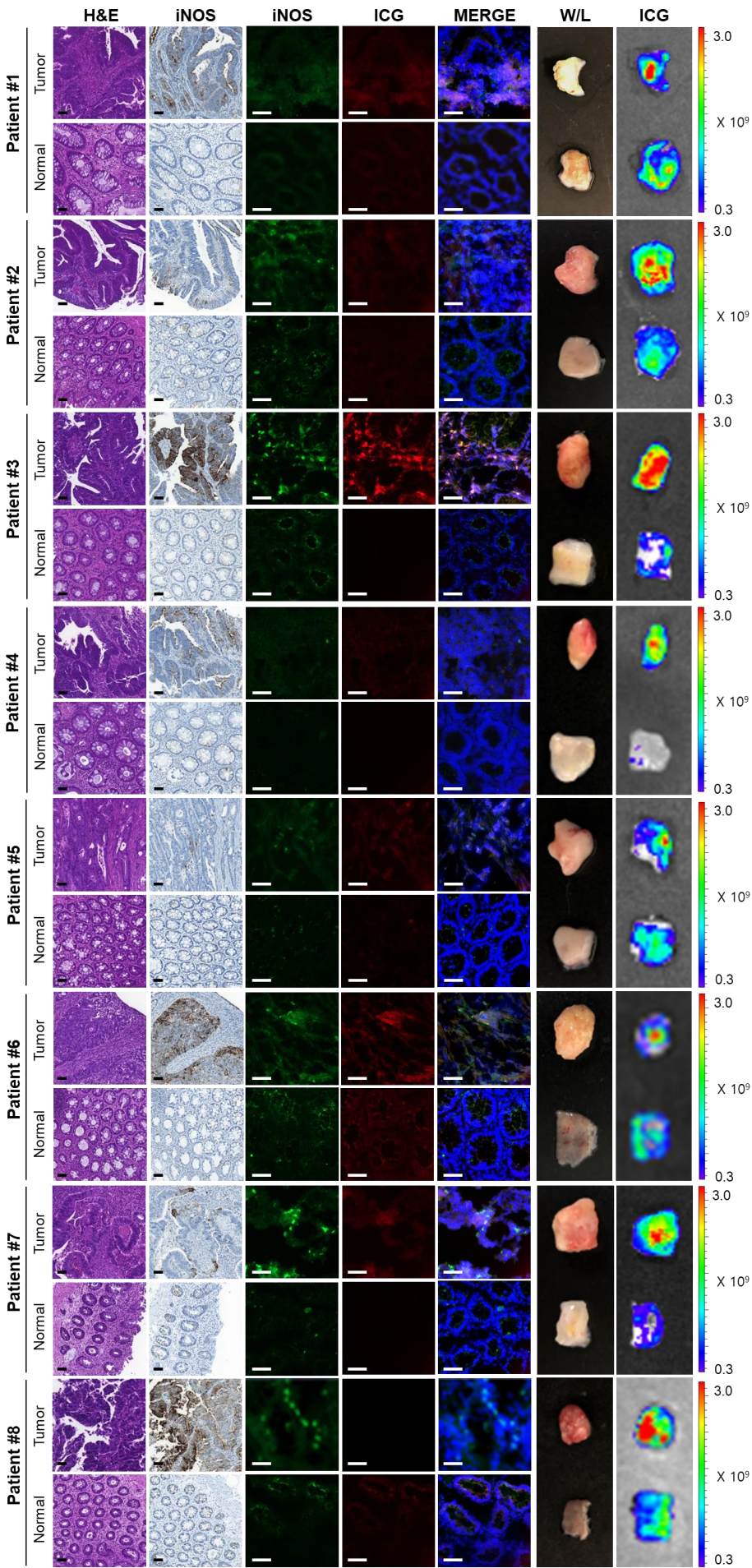

Supplement: S1 File — (ZIP) [file pone.0286189.s001.zip › Supporting information/S6 Fig.pdf]

Supplementary Figure S7.

(A)

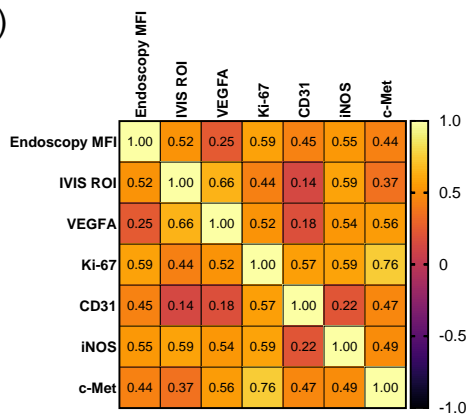

(B)

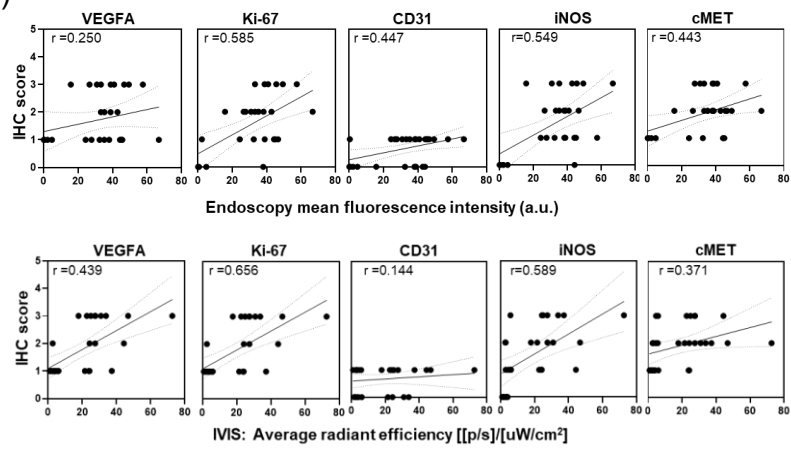

(C)

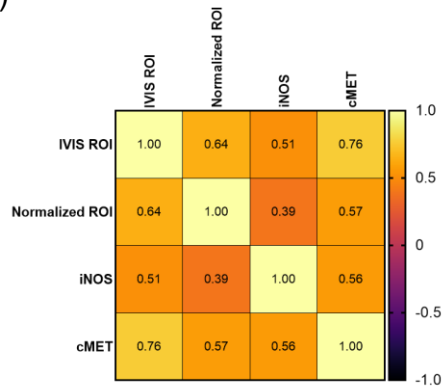

(D)

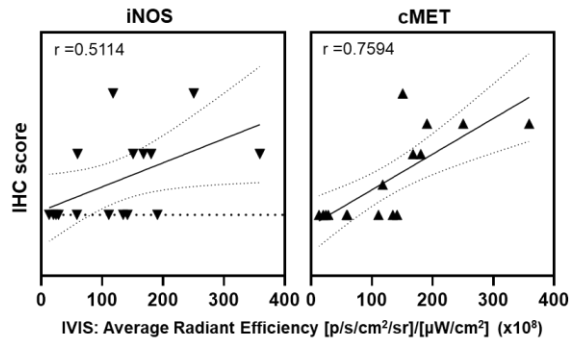

Supplement: S1 File — (ZIP) [file pone.0286189.s001.zip › Supporting information/S7 Fig.pdf]

Supplementary Figure S8.

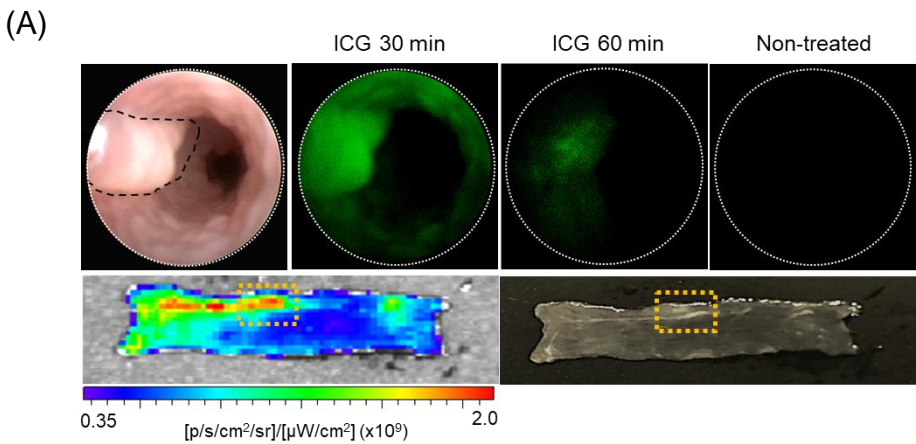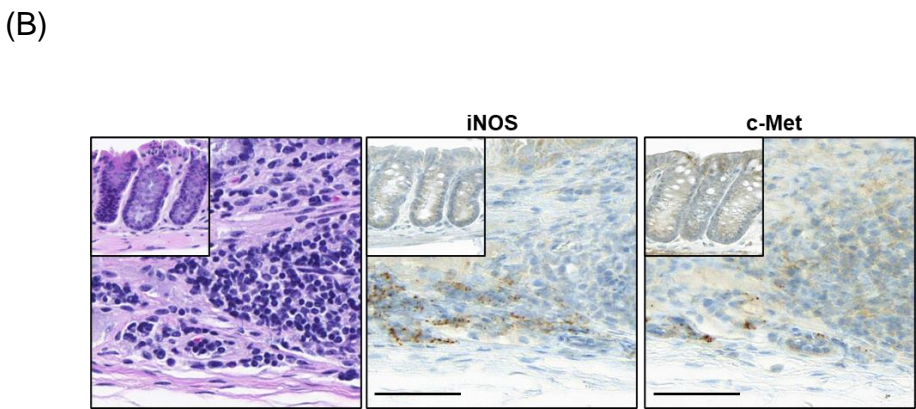

Supplement: S1 File — (ZIP) [file pone.0286189.s001.zip › Supporting information/S8 Fig.pdf]
